# 

**Published:** 1986-02

**Authors:** 


**BRISTOL MEDICO-CHIRURGICAL SOCIETY**

**Contents**

**Editorial**
**Chronic Venous Ulcers of the leg**
S. J. Calder and D. J. Leaper
**Eruptive Vellus Hair Cysts**
C. E. H. Grattan and R. R. M. Harman
**Skins are simpler than you think**
J. L. Burton
**From our Correspondents**
**Bristol Medico-Chirurgical Society**
**From our Foreign Correspondent**
**Opening of the Monica Britton Exhibition Hall**
**Starting to Sail**
A. Roskovec
**Surgical Club of South West England**
**Bristol Medico-Historical Society?**
Proposal to form a new Society
**Letter to the Editor**

